# Early inflammatory macrophage reprogramming by *Scedosporium apiospermum* determines fungal survival

**DOI:** 10.1080/21505594.2026.2707829

**Published:** 2026-09-02

**Authors:** Hélène Guegan, Florence Robert-Gangneux, Sarah Dion, Aymeric Delabarre, Jean-Pierre Gangneux

**Affiliations:** a Université de Rennes, CHU Rennes, INSERM, EHESP, IRSET (Institut de recherche en santé, environnement et travail) - UMR_S, Rennes, France; b CHU Rennes, Laboratoire de Parasitologie-Mycologie, European ECMM Excellence Center in Medical Mycology, Centre National de Référence pour les Mycoses et Antifongiques-Laboratoire Associé Aspergilloses Chroniques, Rennes, France; c Univ Rennes, Inserm, EHESP, IRSET - UMR_S 1085, Rennes, France

**Keywords:** Cytokine, Aspergillus fumigatus, Scedosporium apiospermum, macrophage, cystic fibrosis, transcriptomic profile

## Abstract

*Scedosporium* is the second most prevalent fungal colonizer of the airways of cystic fibrosis (CF) patients after *Aspergillus fumigatus*. Chronic bacterial and fungal colonization drives persistent inflammation via damage-associated molecular patterns, contributing to lung injury. Owing to their distinct structural and biological properties, *S. apiospermum* and *A. fumigatus* may trigger different immune responses and exhibit different susceptibilities to macrophage mediated killing. We compared the transcriptomic and inflammatory profiles of human macrophages challenged with *S. apiospermum* (Sap) or *A. fumigatus* (Afu) for 4 h and 12 h, and assessed fungal survival. Sap activated multiple pro-inflammatory pathways, overlapping with those induced by Afu. At 4 h, 15 inflammation-related pathways were differentially regulated in Sap-infected macrophages (FDR 4 × 10^−2^ to 7 × 10^−13^), 12 of which were also upregulated in Afu-infected cells. The most highly dysregulated pathways involved TNF, IL-17, NF-κB signaling, and cytokine–cytokine receptor interactions. Sap induced a stronger, earlier inflammatory response, with three times more genes upregulated at 4 h than for Afu (671 *vs.* 200), and significantly higher levels of IL-6, IL-1β, TNF-α, IL-23, IL-10 (*p* <0.001), and IFN-α2 (*p* <0.05) secretion. The proportions of macrophages infected at 4 h were similar for the two fungi, but Sap conidia were more efficiently killed after 6 h (46.6% *vs.* 34.5%, *p* <0.05). Thus, *S. apiospermum* elicits a faster and stronger inflammatory macrophage response than *A. fumigatus*, potentially enhancing fungal clearance but also exacerbating airway inflammation in CF.

## Introduction

*Scedosporium apiospermum* is an opportunistic filamentous fungus causing diverse human infections, ranging from colonization or localized infection in immunocompetent hosts to disseminated infections in immunocompromised patients [1]. In the specific context of cystic fibrosis, *Scedosporium* is the second most prevalent fungal colonizer of the airways after *Aspergillus*, being found in 3 to 17% of patients, depending on geographic area [2–4]. Long-term bacterial and fungal airway colonization in CF patients maintains chronic inflammation through the release of various damage-associated molecular patterns (DAMPs) and alarmins, which may be involved in lung injury. In terms of invasive infections, *Scedosporium* has been implicated in both localized (lung, osteoarticular and skin infections) and disseminated infections, although much less frequently than *Aspergillus fumigatus* [5]. A recent French report of 76 *Scedosporium-*related cases over a 13-year period showed that only 26% of infections progressed to disseminated forms, with a mortality for *S. apiospermum* infections of 9% at day 30 [6], far lower than that for invasive aspergillosis, which is generally reported to be up to 70% [7]. However, the outcome of fungal infections also depends strongly on the host, comorbidity and immune system efficacy. The quality of the innate immune response is crucial for fungal clearance, as reflected by the high predisposition to invasive mold infections of patients with immune defects of the myeloid lineage, especially polymorphonuclear cells (PMN) and monocytes. Much of our knowledge of the immune determinants governing the antifungal response has been obtained with models of *A. fumigatus* infection. In lung infections, inhaled fungal conidia cross the epithelial barrier [8] and rapidly encounter first-line immune cells, in the form of resident macrophages and PMNs [9,10]. Macrophages are crucial effector cells in innate immune responses to fungal conidia, as they are the most efficient pathogen scavengers and the predominant source of inflammatory cytokines. Recognition of the surface of the fungal conidia is the first step in the antifungal response, which involves internalization, phagolysosomal acidification, the oxidative burst and fungal killing [9,11]. This early interaction between the fungus and its host involves the binding of pathogen-associated molecular patterns (PAMPs) to various recognition receptors, such as Toll-like receptors (TLR) and C-type lectin receptors on the surface of phagocytes [12].

The availability of PAMPs at the cell surface depends strongly on cell wall composition and the state of the fungus interacting with host cells: dormant conidia or hyphal forms. The conidial cell walls of *A. fumigatus* and *S. apiospermum* have several components in common, including chitin and various polysaccharides, such as alpha- and beta-glucans, which are covered by a layer of melanin, often considered to serve as “armor” protecting the fungus against external aggressions [13–15]. Conidia of both *A. fumigatus* and *S. apiospermum* typically initiate germination within 4 to 6 hours, depending on the experimental conditions [16,17]. During *A. fumigatus* maturation, the conidia undergo a rapid morphological change to an intermediate swollen state before the emergence of the germ tube [16]. *A. fumigatus* conidia are covered by an outer proteinaceous hydrophobin cell-wall layer that disappears during swelling [18] and is not present in *Scedosporium* spp. [19]. Conidial germination is accompanied by the progressive disappearance of melanin, which is not present in hyphae. Immunogenic polysaccharides are, therefore, unmasked in fungal filaments and can be recognized by immune cells, triggering activation and pro-inflammatory responses, as described in *A. fumigatus* [20]. In *Scedosporium* spp., there is little evidence for some modifications of cell-wall surface properties during conidial germination [21], and much less is known about the interaction of the conidia with host phagocyte cells [22].

We hypothesized that differences in cell-wall structure and conidial morphology between these two fungi might affect interactions with host innate immune cells, triggering different inflammatory responses that may ultimately affect fungal survival within the host. We therefore aimed to delineate the macrophage transcriptomic response to *S. apiospermum*, comparing it with the response to *A. fumigatus* infection, and to assess its impact on antifungal pathways.

## Materials and methods

### Fungal strains and preparation of conidia

Both clinical strains used for macrophage infection were kindly provided by the *Fungal respiratory infections* (FRI) team at the university of Angers, France (Prof. JP. Bouchara): i) the *Aspergillus fumigatus* wild-type reference strain Af 293 (Afu), deposited in the National Collection of Pathogenic Fungi (Bristol, UK), under accession number NCPF7367 [23]; and ii) the *Scedosporium apiospermum* wild-type strain (Sap) deposited in the BCCM/IHEM culture collection (Brussels, Belgium) under accession number IHEM 14,462 (WT 14,462) [24].

All experiments were performed with conidia harvested from nine-day-old cultures by scraping, filtered through sterilized Miracloth (pore size 22–25 µm, Sigma-Aldrich, St-Quentin-Fallavier, France) to remove hyphae and washed once in sterile water. Conidia were counted with a LUNA™ automatic particle counter (Logos Biosystems, Villeneuve d’Ascq, France) and conidia suspension were prepared to the required density.

For cytometry and microscopy experiments, conidia were stained with 0.1 mg/mL fluorescein isothiocyanate (FITC, Sigma-Aldrich) in carbonate buffer (0.1 M, pH = 9) at room temperature for 30 min in the dark, washed three times with carbonate buffer and suspended in phosphate-buffered saline (PBS).

### Generation of macrophages

Human peripheral blood mononuclear cells (PBMCs) were isolated from blood buffy coats obtained from healthy donors supplied by *Etablissement Français du Sang*, Rennes, France, as previously described [25,26]. Briefly, PBMCs were isolated by Ficoll-Paque density gradient centrifugation. CD14^+^ cells (monocytes) were isolated by positive magnetic cell-sorting (CD14 Microbeads, Miltenyi Biotec, Paris, France) and M1-polarized macrophages were obtained by incubation in complete medium (RPMI 1640 Gibco, Life Technologies, 10% decomplemented fetal calf serum (FCS), 100 IU/mL penicillin, 100 µg/mL streptomycin) supplemented with 10 ng/mL granulocyte-macrophage colony-stimulating factor (GM-CSF) (Miltenyi, Biotec, Paris, France) for 5 days at 37°C under an atmosphere containing 5% CO_2_.

### Macrophage infection for transcriptomic analysis

A total of 10^6^ differentiated macrophages per well (24-well plates) were inoculated with 1 mL of 10^7^ *A. fumigatus* or *S. apiospermum* conidia suspension per mL, corresponding to a multiplicity of infection (MOI) of 10:1. Plates were centrifuged at 500 × *g* for 5 min at room temperature for 4 h or 12 h at 37°C (under an atmosphere containing 5% CO_2_). The 4 hpi and 12 hpi time points were selected to investigate early and late stages of infection, respectively. At 4 hpi, macrophage uptake is established while *Aspergillus* conidia have not yet initiated swelling or germination; in contrast, *Scedosporium* conidia do not undergo a defined swelling phase. The 12 hpi time point allows the formation of hyphae by both fungi while limiting excessive fungal growth and host cell damage. Replicates were pooled from two donors (four technical replicates each). Uninfected macrophages served as controls. At the end of the incubation period, the supernatant was recovered, the cells were washed twice with PBS, lysed in Macherey-Nagel lysis buffer (Masherey-Nagel, Hoerdt, France) with β-mercaptoethanol, and stored at −80°C.

### RNA sequencing and bioinformatics analysis

RNA extraction, library preparation, sequencing, and bioinformatics analyses were performed by Helixio (Saint-Beauzire, France). Total RNA was extracted with the NucleoSpin RNA kit, and RNA quality and quantity were assessed with NanoDrop, Qubit, and Bioanalyzer machines. Poly(A)-mRNA was isolated, and directional libraries were prepared with NEBNext Ultra II kits. Libraries were subjected to quality checks, quantified, and sequenced (75 bp single reads) on an Illumina NextSeq 500 platform.

Reads were trimmed to remove the adapters (Trimmomatic) and aligned with the human reference genome (GRCh38, Ensembl release 106) with STAR, which was also used to generate gene-count matrices. Quality control was performed with MultiQC. Differential gene expression analysis (edgeR) was performed, comparing *S. apiospermum*-infected and uninfected cells, *A. fumigatus*-infected and uninfected cells, and cells with the two types of infection, at 4 h and 12 h. Genes with an FDR <0.05 (Benjamini–Hochberg correction) and |log_2_FC| >1 were considered to display significant differential expression. Venn diagrams were built with https://www.bioinformatics.com.cn/static/others/jvenn_en/ example.html. Heatmaps were created for the most significantly represented genes of a specific functional class, with the Morpheus tool from https://software.broadinstitute.org/morpheus. A representation of enriched Kyoto Encyclopedia of Genes and Genomes (KEGG) pathways was built with SRplot from https://www.bioinformatics.com.cn.

### Quantitative PCR (qPCR)

RNA-seq results were validated by quantitative real-time PCR analysis for a selected panel of genes differentially expressed between Sap- and Afu-infected cells. RNA was reverse-transcribed to generate cDNA and quantitative PCR amplifications were performed as previously described [27]. The gene-specific primers used are listed in Table S1. Amplification efficiencies were validated, and the levels of transcripts were normalized against the human GAPDH and actin housekeeping genes. Results are expressed as 2–^ΔΔCq^, corresponding to the fold-induction in macrophages infected with *S. apiospermum* relative to the mean quantification cycle obtained with *A. fumigatus*-infected macrophages.

### Phagocytosis assay

Macrophages were plated in 8-well borosilicate Lab-Tek^TM^ plates at a final density of 10^5^ cells/well and infected with FITC-labeled conidia at a MOI of 5:1. Plates were centrifuged at 500 × *g* for 5 min at room temperature to synchronize phagocytosis and were then incubated for 1 h and 4 h at 37°C under an atmosphere containing 5% CO_2_. The cells were then placed on ice and washed twice with cold DPBS to remove non-adherent extracellular conidia. Adherent extracellular conidia were stained with 25 µM calcofluor white (CFW) for 15 min at 4°C. Cells were washed with PBS, fixed with 4% PFA, and observed under a widefield inverted fluorescence microscope (Olympus IX71) equipped with a 20× objective and filters for the detection of FITC (461–489 nm) and CFW (381–399 nm). Images were digitally recorded with SoftWorx software. The proportion of infected macrophages – with FITC^+^ but no CFW^+^ conidia in the cytoplasm – was determined manually, by counting, on at least 1000 cells per condition. The phagocytic index was determined as follows: (total number of engulfed conidia / total number of infected macrophages) × (number of macrophages containing engulfed conidia / total number of macrophages counted) × 100.

### Killing assay

Macrophages were plated at 5 × 10^5^/well and infected with FITC-labeled conidia at a MOI of 1:1. Phagocytosis was synchronized as described above and plates were incubated at 37°C under an atmosphere containing 5% CO_2_, in the dark, for 6 h. Plates were then placed on ice, and the cells were washed three times with cold DPBS and immediately lysed with cold sterile water. They were then incubated at 4°C for at least 1 h or overnight. The plates were centrifuged at 10,000 × *g* for 5 min and the pelleted conidia were stained by incubation with 25 µg/mL propidium iodide (PI, Sigma-Aldrich) for 30 min at room temperature in the dark. Conidia were washed with sterile water, fixed with 4% PFA and analyzed with a LSR Fortessa X20 cytometer (BD Biosciences, Le-Pont-de-Claix, France). Each replicate included a negative control, corresponding to live conidia (unexposed FITC^+^ conidia) and a positive control corresponding to heat-killed conidia (85°C, 20 min), used to set gates for the positivity of each marker. Live conidia were FITC^+^ PI^−^ and dead conidia were double-positive (FITC^+^ PI^+^). The percent live conidia was determined as the percentage of PI^−^ conidia among FITC^+^ conidia. The decrease in conidial survival was calculated relative to control conidia not exposed to macrophages (viability control) as: 100*((% live conidia among unexposed conidia - % live conidia among cell-exposed conidia) / % live conidia among unexposed conidia).

### Cytokine assays

Macrophages were plated at a density of 10^6^ cells/well and incubated with 10^7^ live conidia (MOI 10:1) in complete RPMI for 12 h at 37°C under an atmosphere containing 5% CO_2_. Supernatants were collected and immediately frozen at −80°C for storage. Pro-inflammatory cytokines were determined in a 13-plex bead-based immunoassay, the LEGENDplex® multi-analyte flow assay (Human Inflammation Panel 1) (BioLegend, Paris, France), performed according to the manufacturer’s instructions, on an LSR Fortessa X20 cytometer (BD Biosciences, Le-Pont-de-Claix, France). The data were processed on the online platform provided by the kit manufacturer.

### Oxidative stress (reactive oxygen species, ROS) assay

Macrophages were plated at a density of 2 × 10^5^ cells per well in 96-well plates and were incubated with 2 × 10^6^ live *S. apiospermum* or *A. fumigatus* conidia (MOI 10:1) in complete RPMI for 2 h at 37°C under an atmosphere containing 5% CO_2._ A control stimulated with 10 µM phorbol-12-myristate-13-acetate (PMA) and a negative untreated control were included. Cells were washed with PBS and the probe (10 µM CM-H_2_DCFDA in PBS, Thermo Fisher Scientific, Les Ulis, France) was added. Plates were incubated at 37°C under an atmosphere containing 5% CO_2_ for 3 h and oxidation of the probe was detected as an increase in fluorescence at 485–520 nm on a CLARIOstar Plus (BMG LABTECH, Champigny-sur-Marne, France) microplate reader. Results are expressed as the fluorescence of infected or treated macrophages divided by the fluorescence of untreated macrophages.

### Statistical analysis

All experiments were performed in triplicate or quadruplicate, at least three times independently, except for RNA sequencing (two experiments). Data are expressed as the mean ± standard error of the mean (SEM). Statistical analysis was performed with GraphPad Prism v.8 software (GraphPad Software, Inc., La Jolla, CA, USA). A one-way analysis of variance, Kruskal–Wallis test or Mann–Whitney U test was performed to evaluate differences between Sap- and Afu-infected macrophages, and between infected and uninfected macrophages. In all analyses, *p*-values below 0.05 were considered statistically significant.

## Results

RNA sequencing was performed on human primary macrophages infected with either *S. apiospermum* (Sap) or *A. fumigatus* (Afu) conidia. Changes in gene expression after 4 h (early infection) and 12 h (late infection) were investigated.

### *S.* *apiospermum* and *A.* *fumigatus* trigger the activation of similar inflammatory pathways in human macrophages

We first investigated the transcriptomic profile of macrophages in response to i) *S. apiospermum* (Sap) and ii) *A. fumigatus* (Afu), with uninfected cells as the control. Following Sap infection, 1,169 and 3,852 differentially expressed genes (DEGs) were identified at 4 h and 12 h postinfection (pi), respectively, with DEGs defined as genes with an FDR <0.05 and an absolute log_2_fold change (FC) in expression >1 (Figure S1a, b). The proportion of upregulated genes was 57.4% (671/1,169) at 4 h, decreasing to 45.7% (1,759/3,852) at 12 h pi. A similar trend was observed when the analysis was restricted to genes with a│log_2_FC│>2, with 85.5% (189/221) and 59.3% (644/1,085) of genes upregulated at 4 h and 12 h, respectively (Figure S1b). Interestingly, only 32.6% (597/1,833) and 17.3% (383/2,208) of genes were differentially up- and downregulated, respectively, at both time points (Figure S1c).

By contrast, Afu infection triggered the differential expression of only 256 genes at 4 h, with a considerable increase to 1,996 genes at 12 h (Figure S2a, b). The proportion of upregulated genes was 78.1% and 59.4 at 4 h and 12 h, respectively. The most strongly differentially expressed genes (│log_2_FC│>2) were predominantly upregulated, accounting for 92.6% (50/54) and 88.0% (500/568) of DEGs at 4 h and 12 h, respectively (Figure S2b). Only 4.6% (38/829) and 16.6% (197/1,188) of DEGs were common to the 4 h and 12 h time points, for down- and upregulated genes, respectively (Figure S2c).

Based on the KEGG database, the various DEGs identified during Sap and Afu infections were grouped into functional categories, presented in Table 1. At 4 h pi, 15 pathways involved in inflammatory processes were differentially expressed in Sap-infected macrophages (FDR of 4 × 10^−2^ to 7 × 10^−13^), 12 of which were also differentially expressed in Afu-infected macrophages (FDR of 4 × 10^−2^ to 0), regardless of the direction of regulation (down- or upregulated). The four most strongly dysregulated pathways were signaling pathways associated with TNF, IL-17, NF-kappa B, and actors of cytokine–cytokine receptor interactions, such as CXCL2, CCL3L1 and CCL4, commonly described during antifungal responses. At 4 h pi, an enrichment in four additional pathways involved in cell-cycle regulation or programmed cell death was detected in Sap-infected macrophages, but not in Afu-infected cells. In Sap-infected macrophages, the pathways significantly modified were essentially similar at 12 h and at 4 h, with the exception of the RIG-I-like receptor pathway, and Th1, Th17 and Th17 cell differentiation, for which no enrichment was detected at 12 h. In addition to phagosome-related pathways, significant enrichment was detected for the PI3K-Akt and ferroptosis pathways at 12 h in Sap-infected macrophages, but not in Afu-infected macrophages.

**Table 1.** Summary of pathways for which enrichment was detected in Sap-infected or in Afu-infected macrophages relative to uninfected macrophages, at 4 h and 12 h postinfection.

KEGG Pathway		FDR			
		Sap *vs*. uninfected		Afu *vs*. uninfected	
Identifier	Description	4 h	12 h	4 h	12 h
hsa04668	TNF signaling pathway	1E-12	5E-05	4E-12	1E-11
hsa04657	IL-17 signaling pathway	1E-06	3E-04	3E-11	2E-04
hsa04060	Cytokine-cytokine receptor interaction	7E-13	8E-08	0	3E-12
hsa04062	Chemokine signaling pathway	2E-05	6E-06	4E-06	9E-07
hsa04620	Toll-like receptor signaling pathway	1E-06	1E-03	1E-07	2E-08
hsa04625	C-type lectin receptor signaling pathway	8E-05	6E-04	8E-05	1E-05
hsa04621	NOD-like receptor signaling pathway	8E-07	8E-03	3E-05	3E-06
hsa04630	JAK-STAT signaling pathway	3E-04	7E-03	1E-04	1E-04
hsa04010	MAPK signaling pathway	1E-03	8E-04	5E-03	3E-04
hsa04064	NF-kappa B signaling pathway	3E-09	2E-06	4E-10	4E-08
hsa04066	HIF-1 signaling pathway	2E-03	2E-03	X	6E-03
hsa04622	RIG-I-like receptor signaling pathway	2E-02	X	3E-02	X
hsa04657	Th17 cell differentiation	2E-02	X	4E-02	1E-02
hsa04660	T-cell receptor signaling pathway	4E-03	4E-02	X	X
hsa04658	Th1 and Th2 cell differentiation	4E-02	X	X	6E-03
hsa04145	Phagosome	X	4E-02	X	X
hsa04110	Cell cycle	4E-03	4E-07	X	3E-03
hsa04218	Cellular senescence	4E-03	7E-06	X	5E-03
hsa04210	Apoptosis	7E-04	7E-03	X	2E-03
hsa04068	FoxO signaling pathway	2E-02	6E-04	X	X
hsa04151	PI3K-Akt signaling pathway	X	4E-02	X	X
hsa04216	Ferroptosis	X	6E-03	X	X

FDR: false discovery rate indicating the significance of pathway enrichment. “X” indicates that the pathway is not significantly represented in the given condition. Pathways referring to specific diseases, e.g. “Influenza A,” “legionellosis” or “rheumatoid arthritis” are not shown.

**Figure 1.**
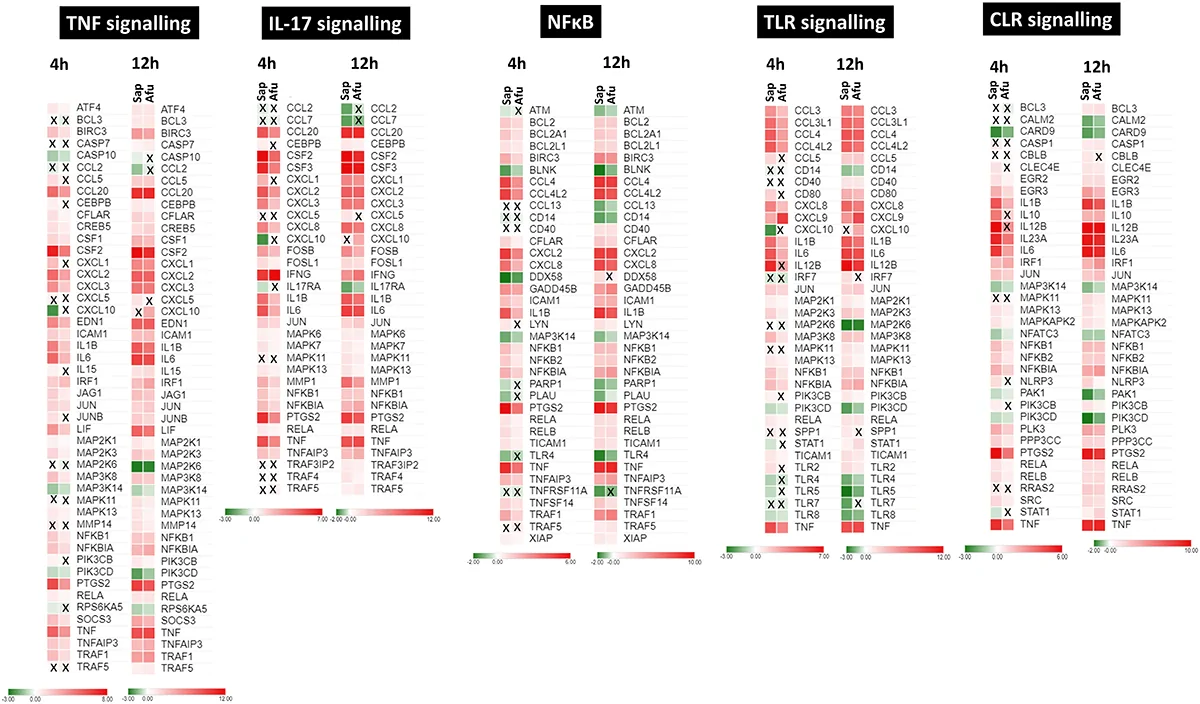
Heatmaps of five pathways displaying significant enrichment in Sap- and Afu-infected macrophages relative to uninfected macrophages at 4 h and 12 h postinfection. The color scale represents log_2_FC values, from dark green (negative values, downregulated genes) to dark red (positive values, upregulated genes). The intensity of the color reflects the magnitude of the change in gene expression. Genes are indicated if significant differences in their expression (FDR <0.05) were found in at least one of the four comparisons, and “X” indicates non-significance (FDR >0.05). DEGs common to the represented pathways were not removed.

We investigated these pathways further by depicting the fold-change in the expression of DEGs displaying significant changes in Sap- or Afu-infected macrophages on heatmaps (Figure 1). Five major pathways involved in antifungal immunity were identified. Interestingly, many genes involved in inflammation, mostly encoding cytokines and chemokines, were upregulated in response to Sap or Afu infection. For instance, 87.2% (35/40) and 87.2% (41/47) of the DEGs corresponding to the TNF signaling pathway were upregulated in Sap-infected macrophages relative to uninfected macrophages, at 4 h and 12 h, respectively. Similarly, 90.6% (29/32) and 91.1% (41/45) of the DEGs in Afu-infected macrophages were upregulated at 4 h and 12 h, respectively. The direction of regulation was conserved between 4 h and 12 h pi.

The fold-change in expression for upregulated DEGs was generally greater at 12 h than at 4 h, except for the IFN-γ gene (associated with IL-17 signaling), the induction of which remained stable over time (FC 5.7 vs 5.4 in Sap–infected *vs*. uninfected macrophages and 7.0 *vs.* 6.8 in Afu–infected *vs.* uninfected macrophages at 4 h and 12 h, respectively).

### *S.* *apiospermum* induces an earlier, stronger, pro-inflammatory transcriptomicresponse than *A.* *fumigatus*

**Figure 2.**
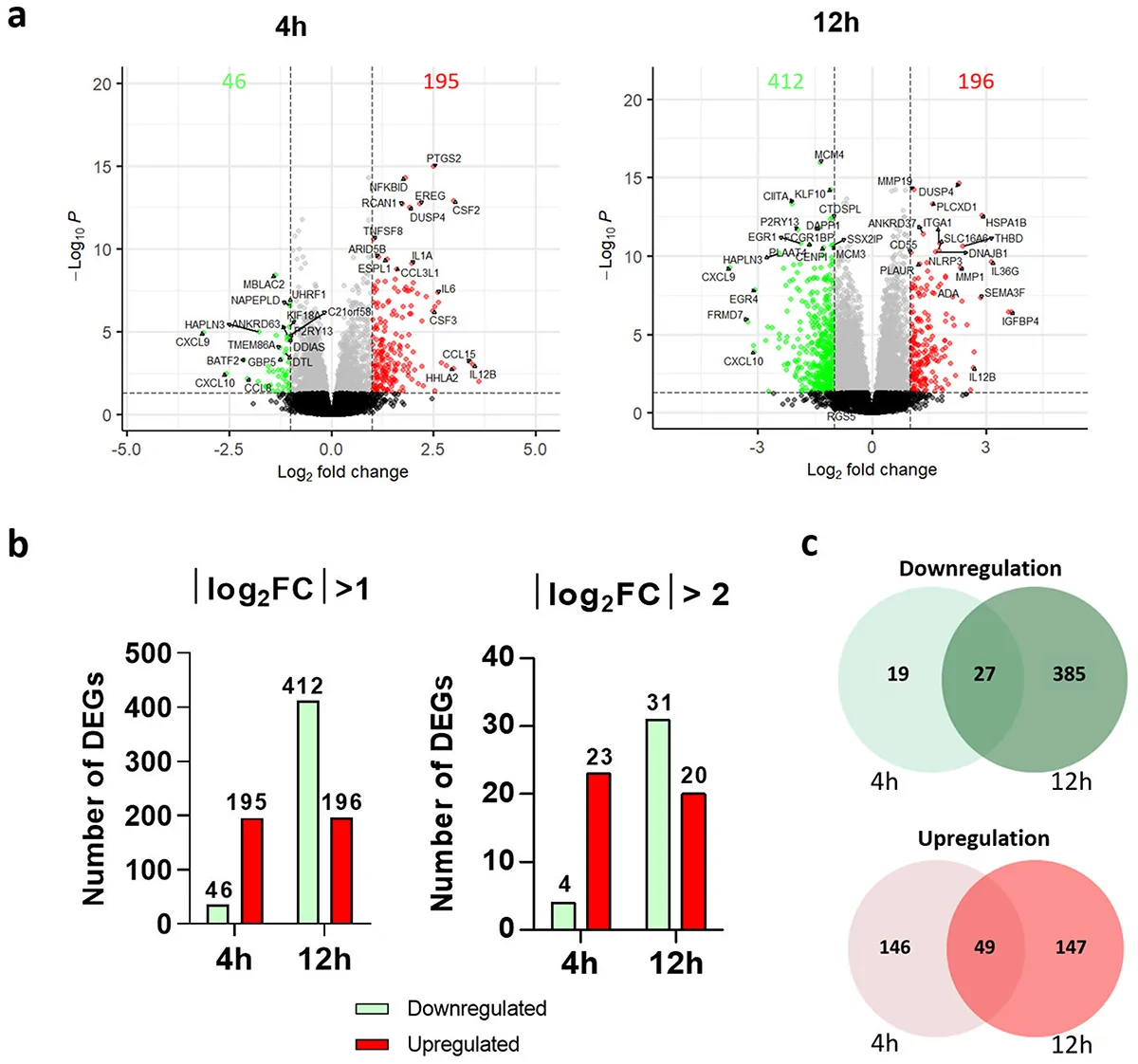
Genes differentially expressed between *S. apiospermum*-infected macrophages and *A. fumigatus*-infected macrophages. (a) Volcano plots of DEGs at 4 h and 12 h pi. Green and red dots represent significant DEGs (FDR <0.05) that are downregulated (log_2_FC <−1) and upregulated (log_2_FC >1), respectively. Light gray and black dots correspond to genes with |log_2_FC |<1 or FDR >0.05. The gene IDs of some of the most highly dysregulated genes are shown. (b) Histograms showing the number of DEGs at 4 h and 12 h, for thresholds of |log_2_FC|>1 (left) or >2 (right). (c) Venn diagrams showing the number of genes downregulated (top) or upregulated (bottom) specifically at 4 h or 12 h, or at both time points during infection (|log_2_FC| >1).

As Sap and Afu appeared to stimulate similar pathways, we further compared the intensity and kinetics of the macrophage responses triggered by these fungi. We re-analyzed the data, focusing on the profile of differential expression between Sap-infected macrophages and Afu-infected macrophages, rather than uninfected macrophages. There were 241 DEGs and 608 DEGs at 4 h and 12 h pi (Figure 2(a)). Interestingly, at 4 h pi, upregulated genes were more predominant in Sap-infected than in Afu-infected macrophages, accounting for 80.9% (195/241) *vs.* 32.2% (196/608) of DEGs. By contrast, downregulated genes predominated at 12 h, accounting for 67.8% (412/608) of DEGs (Figure 2(b), left). A similar trend was observed for the most strongly differentially regulated genes (|log_2_FC|>2), with a stable number of upregulated genes (23 at 4 h and 20 at 12 h) and increasing number of downregulated genes, from 4 at 4 h to 31 at 12 h (Figure 2(b), right). The gene ID, log_2_FC and FDR of these most strongly DEGs are presented in Table S2. Only 27/431 (6.3%) downregulated DEGs and 49/342 (14.3%) upregulated DEGs were common to the two time points (Figure 2(c)).

**Figure 3.**
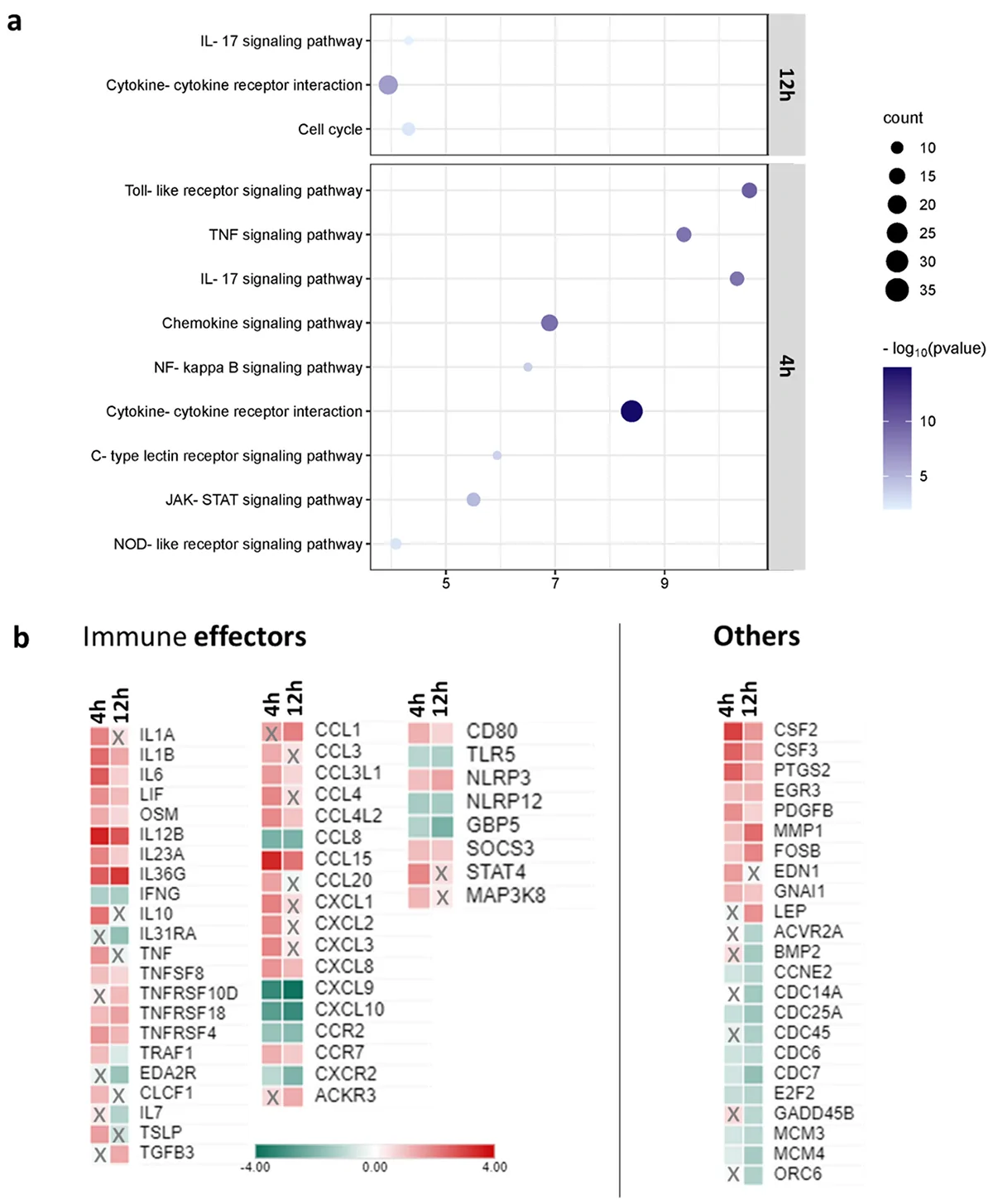
Differential gene expression profiles of macrophages infected with *S. apiospermum* conidia and macrophages infected with *A. fumigatus* conidia, at 4 h and 12 h postinfection. (a) KEGG pathways for which enrichment was detected in Sap-infected cells relative to Afu-infected cells. Only enrichments with a FDR <0.05 are shown. (b) Heatmaps of DEGs from the 10 enriched pathways (72 genes) at 4 h and/or 12 h shown in (a). The color scale represents log_2_FC values, from dark green (negative values, downregulated genes) to dark red (positive values, upregulated genes). The intensity of the color reflects the magnitude of the change in gene expression. Genes are indicated if their expression had changed significantly (FDR <0.05) at 4 h or 12 h, and “X” indicates an absence of significance (FDR >0.05).

There were fewer DEGs in Sap-infected macrophages at 4 h and 12 h, but the number of significantly enriched pathways at 4 h exceeded the number of enriched pathways at 12 h (9 *vs.* 3) (Figure 3(a)). The most represented DEGs corresponded to inflammatory process pathways (Toll-like receptors, TNF and IL-17) and cytokine–cytokine receptor interactions. The other pathways represented included various PRRs (C-type lectin and NOD-like receptors) and members of the NF-kB and JAK-STAT signaling pathways also known to be associated with antifungal immunity. All DEGs from the 10 enriched pathways at any time point (Figure 3(a)) are represented on heatmaps showing the change in gene expression (Figure 3(b)). We found that 48 of the 72 (63.9%) non-redundant genes differentially expressed at 4 h and/or 12 h were involved in antifungal immunity. These genes encoded cytokines (*n* = 15) and cytokine receptors (*n* = 7), chemokines (*n* = 15) and chemokine receptors (*n* = 4), inflammasome proteins (*n* = 2), signaling proteins (*n* = 3), TLR (*n* = 1) and the differentiation cluster CD80. In total, 32 of the 41 DEGs at 4 h and 22 of the 30 DEGs at 12 h were upregulated in Sap-infected macrophages. Most pro-inflammatory cytokines and chemokines were upregulated at both time points, but with generally greater fold-changes in expression at 4 h than at 12 h. Specifically, a panel of genes encoding crucial pro-inflammatory cytokines from the IL-1 family (IL-1α, IL-1β, IL-36γ) and the IL-6 family (IL-6, LIF, OSM), as well as IL-12 p40 subunit (*IL12B*) and IL-23 p19 subunit *(IL23A*) were strongly upregulated in Sap-infected cells relative to Afu-infected cells, predominantly at 4 h pi. In addition, genes encoding the PMN chemoattractants CCL15, CXCL1, CXCL2, CXCL3 and CXCL8 (log_2_FC from 1.68 to 3.40, FDR 7 × 10^−3^ to 2.7 × 10^−8^) were significantly overexpressed in Sap-infected macrophages relative to Afu-infected macrophages. By contrast, the pleiotropic chemokine CCL8, together with CXCL9 and CXCL10, which act on activated T cells and NK cells, were downregulated in Sap-infected cells relative to Afu-infected cells, at both 4 h and 12 h. The other genes downregulated in Sap-infected macrophages encoded various receptors, such as IL-1RA, CCR2, CXCR2 and TLR5, and the inflammasome regulatory protein NLRP12. Most of the other DEGs in Sap-infected cells encoded growth factors, such as CSF2, CSF3 and PTGS2 (overexpressed), and various cell cycle regulators (mostly downregulated).

We selected a panel of genes for RT-qPCR studies to validate the RNA-seq data; the results obtained were consistent with a strong induction of genes encoding pro-inflammatory effectors, such as IL-1β, IL-6, TNF and CCL15 (Figure S3).

### Sap-infected macrophages release larger amounts of inflammatory cytokines than Afu-infected macrophages

**Figure 4.**
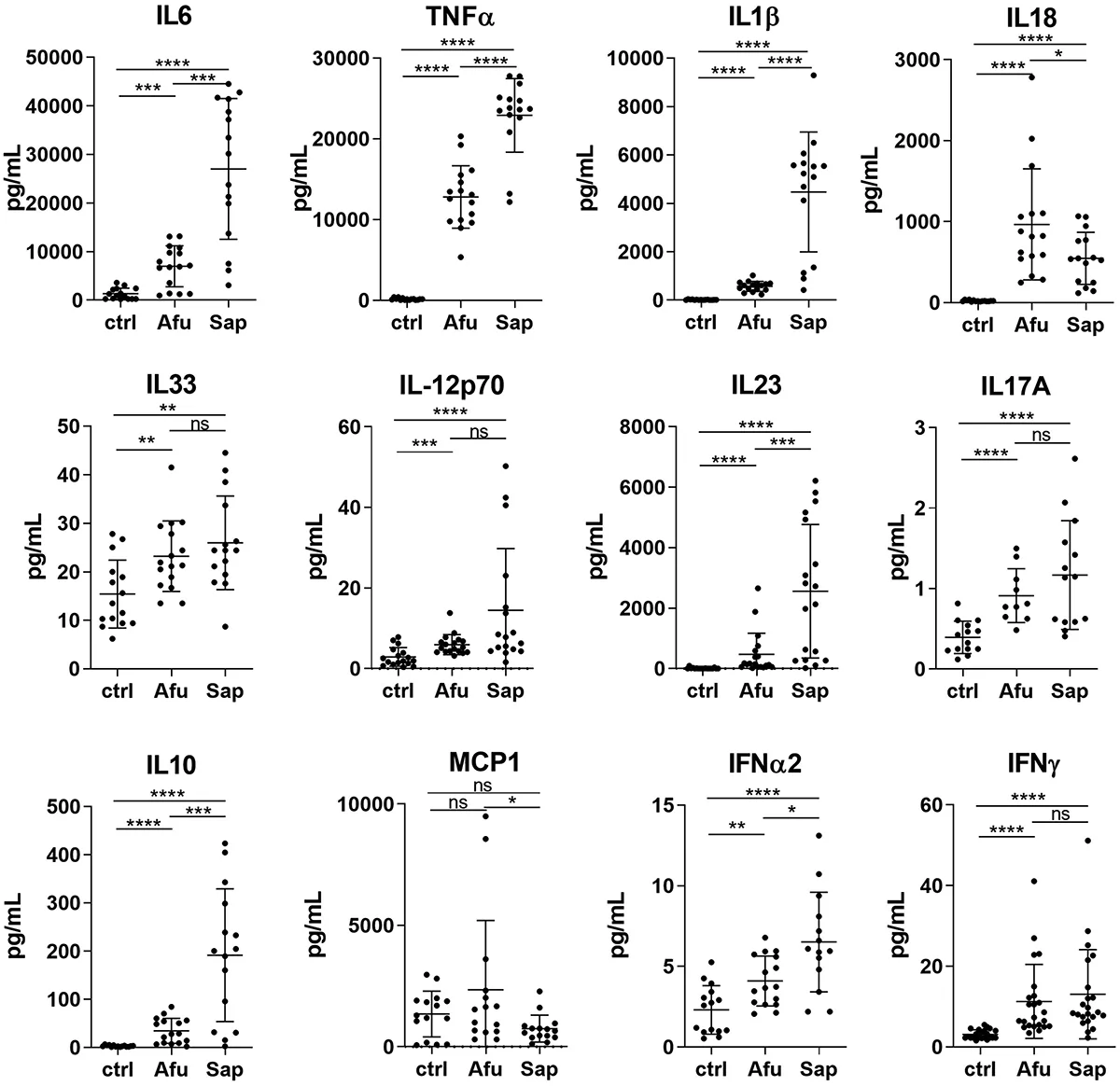
Concentration of cytokines and chemokines in the supernatants of macrophages infected with *S. apiospermum* or *A. fumigatus*, 12 h postinfection. Uninfected macrophages were included as controls (ctrl). Determinations were performed in a 13-plex bead-based immunoassay, the LEGENDplex® multi-analyte flow assay (BioLegend). *p-value <0.05; **p-value <0.01; ***p-value <0.001; ****p-value <0.0001; Mann-Whitney *U* test.

We further validated the exacerbated inflammatory profile of Sap-challenged macrophages relative to Afu-challenged macrophages by determining the levels of cytokines and chemokines in the supernatants of 12-hour cultures (Figure 4). Interestingly, Sap infection triggered the release of larger amounts of IL-6, IL-1β, TNF-α, IL-23, IL-10 (*p* <0.001) and IFN-α2 (*p* <0.05) than Afu infection, in accordance with the transcriptomic results. By contrast, levels of IL-18 and MCP1 (CCL2) were higher in supernatants from Afu-infected macrophages than in those from Sap-infected macrophages.

### Efficacy of macrophage effector functions against Sap and Afu conidia

**Figure 5.**
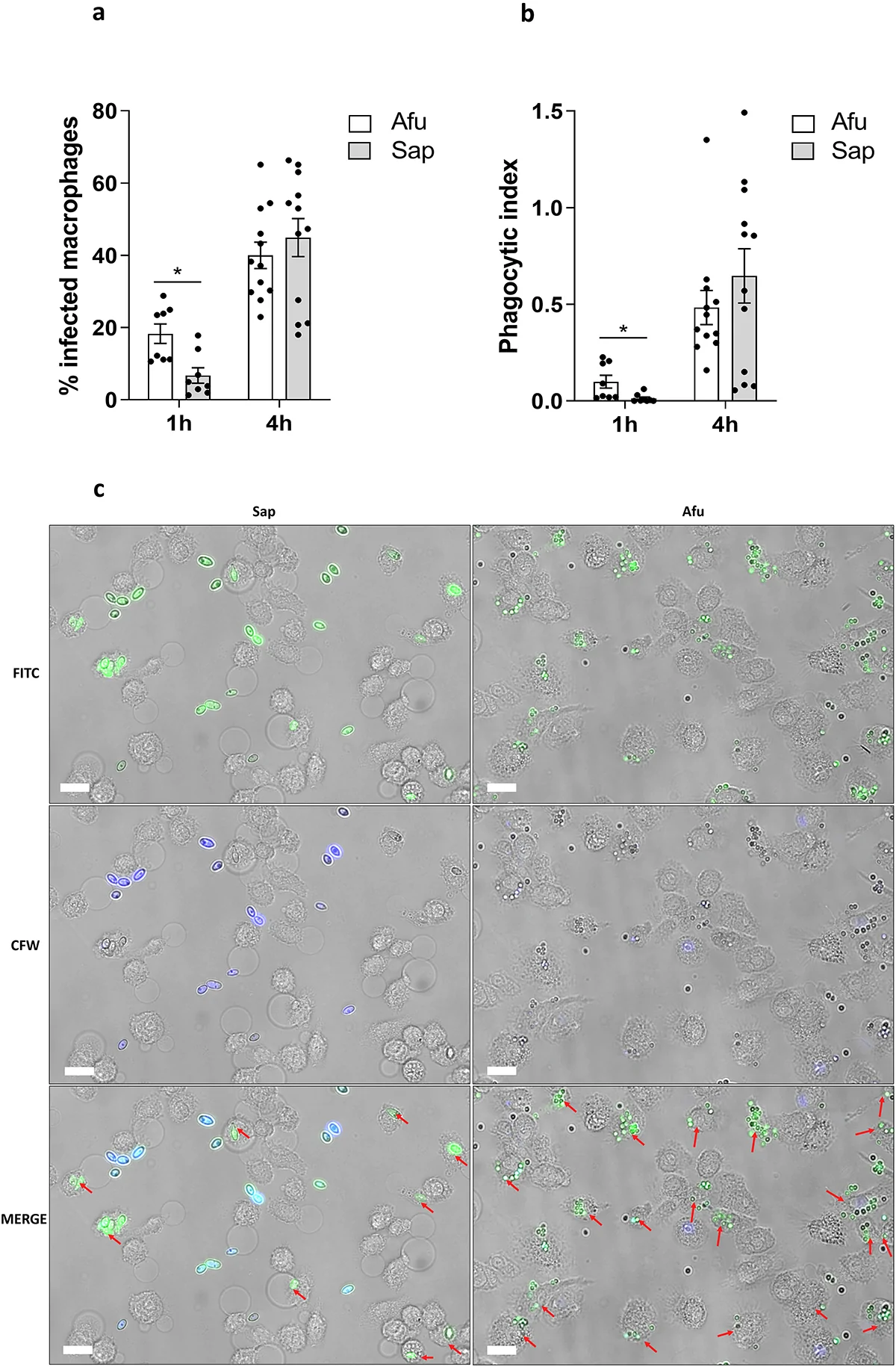
Phagocytosis of *S. apiospermum* (Sap) and *A. fumigatus* (Afu) conidia by macrophages. (a) Percentage of infected macrophages following incubation at 37°C under an atmosphere containing 5% CO_2_ for 1 h or 4 h, (MOI 5:1). Counting based on microscopic slide scanning. (b) Phagocytic index at 1 h and 4 h pi. (c) Example of microscopic acquisition from macrophage cultures infected with FITC-labelled Sap and Afu conidia (shown in green) for 1 h at 37°C under an atmosphere containing 5% CO_2_. Counterstaining with calcofluor white (CFW) was used to stain extracellular conidia (shown in blue). Scale bars: 20 µm. Red arrows indicate conidia‑containing macrophages. *p-value <0.05, Mann-Whitney *U* test.

The recognition of extracellular fungal spores and the subsequent activation of inflammation pathways in phagocytes are thought to contribute to the successful engulfment and killing of pathogens. We therefore compared the phagocytosis of Sap and Afu conidia by macrophages at 1 h and 4 h pi. Afu conidia were internalized slightly more rapidly than Sap conidia, as illustrated by the higher rate of infected macrophages (29.2 ± 15.4% *vs.* 25.8 ± 19.1%, *p* = 0.010), and the higher phagocytic index (0.1 *vs.* 0.01, *p* = 0.010) after 1 h of incubation (Figure 5(a–c)). The proportions of Sap- and Afu-infected macrophages were also similar at 4 h (Figure 5(a)).

**Figure 6.**
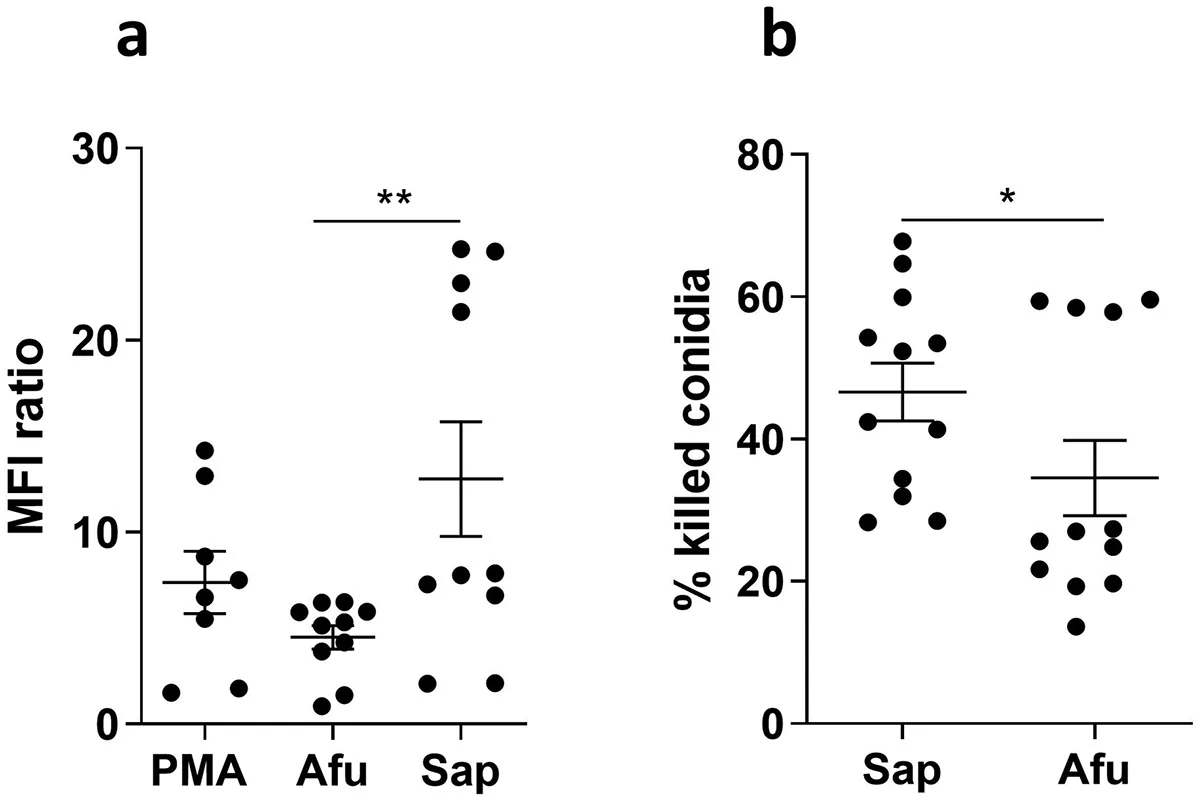
Evaluation of phagocytic functions (a) Reactive oxygen species (ROS) assay. Macrophages were incubated with *S. apiospermum* or *A. fumigatus* conidia at 37°C under an atmosphere containing 5% CO_2_ for 2 h (MOI 10:1). The cells were washed with PBS and then incubated for 3 h with the CM-H_2_DCFDA probe. The fluorescence emitted by the CM-H_2_DCFDA probe after hydrolysis by intracellular oxidative metabolism in macrophages was measured at 485–520 nm. Data are presented as the ratio of raw fluorescence between infected and uninfected macrophages. Phorbol-12-myristate-13-acetate (PMA)-treated cells were used as positive control. (b) Conidial killing after ingestion by macrophages. After incubation for 6 h (MOI 1:1), extracellular conidia were removed by three washes with PBS, and internalized conidia were released by macrophage lysis (cold water) for the viability assay. Killing rates were determined relative to control conidia not exposed to macrophages (viability control) as follows: 100*((% live conidia among unexposed conidia − % live conidia among cell-exposed conidia) / % live conidia among unexposed conidia).*p-value <0.05, Mann-Whitney *U* test.

The internalization of conidia after infection with either Afu or Sap was accompanied by the activation of ROS production (Figure 6(a)). However, Sap triggered a higher level of fluorescence when infected macrophages were incubated with the detection probe, reflecting higher levels of ROS production in Sap- than in Afu-infected cells.

Finally, we evaluated the killing of Sap and Afu conidia by macrophages after 6 h of co-incubation. We found that Sap was less able to survive the antifungal mechanisms of macrophages, as 46.6% and 34.5% of Sap and Afu conidia, respectively, were killed (Figure 6(b), *p* <0.05).

## Discussion

The macrophage response to *S. apiospermum* conidia remains poorly explored. Current knowledge of the determinants of the antifungal immune response was mostly extrapolated from studies focusing on a small number of human fungal pathogens, principally *A. fumigatus*, followed by *Candida albicans* and *Cryptococcus neoformans* [9,28–30]. We report here the first comparative transcriptomic analysis of human macrophages challenged with *S. apiospermum* conidia or *A. fumigatus* conidia, at two different time points: 4 h and 12 h pi.

We first showed that *S. apiospermum* shaped the activation of several pro-inflammatory pathways similar to those activated by A. *fumigatus*. Following the recognition of fungal patterns by CLRs and TLRs, activation of the NFκB pathway is the mainstay of innate immunity and crucial for the clearance infection, as widely reported for both *A. fumigatus* [31] and *Scedosporium* [32]. The inflammatory cascade supported by the release of large amounts of IL-6 and TNF-α into culture supernatants was expected, as these cytokines play an important role in antifungal immunity, stimulating neutrophil migration and inducing the oxidative burst in phagocytes [33]. Another crucial inflammatory pathway is that of inflammasome activation, involving multiprotein platforms that activate caspase-1, which is ultimately essential for the cleavage of pro‑IL-1β and pro‑IL-18 to generate their mature, biologically active forms [34,35]. The upregulation of the *NLRP3* gene, together with the downregulation of the negative regulator *NLRP12* in *S. apiospermum*-infected cells may be associated with the release of large amounts of IL-1β into cell supernatants. By contrast, the reasons for the release of larger amounts of IL-18 released by *A. fumigatus*-infected cells remain to be elucidated, as this cytokine is activated concomitantly with the processing of IL-1β. Both IL-1β and IL-18 are known to initiate Th1 and Th17 adaptive immune responses [36]. Here, both *S. apiospermum* and *A. fumigatus* were found to trigger activation of IL-17 pathway, which is generally considered beneficial for fungal clearance, although several contradictory reports have been published [37].

Strikingly, we observed that the activation of pro-inflammatory pathways was stronger and occurred earlier for *S. apiospermum*, which induced the overexpression of three times as many DEGs at 4 h as *A. fumigatus* (671 *vs.* 200), after independent normalization on uninfected cells. These findings were further supported by the expression analysis on *S. apiospermum*–infected cells using *A.fumigatus*-infected cells as the comparator, which showed that 80% of differentially expressed genes were upregulated in Sap-infected cells at 4 h. At 12 h, *A. fumigatus* infection triggered an inflammatory transcriptomic profile essentially similar to that triggered by *S. apiospermum*, but with a number of pro-inflammatory cytokine-related genes remaining downregulated, relative to S. *apiospermum*-infected macrophages. These observations suggest that *A. fumigatus* conidia were recognized later than those of *S. apiospermum*. Importantly, concomitant ROS induction was lower and the survival of conidia was higher in *A. fumigatus*-infected than in *S. apiospermum*-infected cells.

Several discrepancies regarding *CXCL9* and *CXCL10* were observed, with these two genes unexpectedly downregulated in *S. apiospermum-* relative to *A. fumigatus*-infected macrophages, and even repressed relative to uninfected cells. This observation requires confirmation and further investigations are required, given the importance of these chemokines for antifungal clearance. Indeed, the protective role of these CXCR3-binding chemokines against fungal infections is now widely accepted, as polymorphisms of *CXCL10* have been implicated in the susceptibility of hematopoietic stem-cell transplant patients to invasive aspergillosis [38].

At first glance, this finding contrasts with a previous report for the *S. boydii* model, showing a strong release of CXCL10, along with TNF, IL-6 and IL-10, by mouse peritoneal macrophages [32]. However, the macrophage response may also vary with the model used, i.e. human *vs.* murine macrophage cells, and according to the fungal species studied. In this regard, several differences in cell-wall composition and biofilm-forming capacity have been described between *Scedosporium* species [14,39–41], along with their association with pathogenicity in the *Galleria mellonella* model [42]. One recent study characterized activation of the macrophage inflammatory response following infection with two *Scedosporium* species, highlighting the induction of a stronger and earlier inflammatory response by *S. dehoogi* than by *S. apiospermum* at 3 h pi [43]. This study is the first to compare macrophage responses to *S. apiospermum* and *A. fumigatus*, but other studies have compared *Lomentospora prolificans*, which was previously classified as a member of the genus *Scedosporium*, to *Aspergillus* species. Interestingly, *L. prolificans* also induced the synthesis of higher levels of TNF-α and IL-6 by human monocytes *in vitro* [44]. The differential inflammatory and antifungal responses observed in this study were assessed at two time points chosen to represent early and late stages of the host–pathogen interaction. We recognize that both the selection of these time points and the multiplicity of infection employed may have influenced the magnitude and nature of the responses observed.

Several hypotheses relating to cell-wall composition can be put forward to explain the triggering of a stronger inflammatory macrophage by S. *apiospermum* than by *A. fumigatus*. The preliminary step in the interaction between the fungus and the host cell is the attachment of the conidia to the host cell, which is highly dependent on the adhesive properties of the fungal conidia [45]. We show here that the internalization of S. *apiospermum* conidia in macrophages is comparable to that of *A. fumigatus* conidia, except that it occurs slightly later. The cell walls of *Scedosporium* conidia are smooth, with no particular organization, whereas those of *A. fumigatus* conidia are entirely covered by rodlets [46]. This textured surface renders the dormant conidia highly hydrophobic, with a strong electronegative surface charge, enabling the conidia to bind host to extracellular matrix proteins, such as laminin and fibronectin [47–49]. Although high conidial surface hydrophobicity and a strongly electronegative zeta potential have been reported among *Scedosporium* species, *S. apiospermum* conidia appear to exhibit relatively low hydrophobicity, likely lower than that reported for *A. fumigatus* [19,39,46]. Moreover, conidial hydrophobicity is a highly dynamic property that varies with the maturation stage and markedly decreases during the germination process [39,46]. A direct comparison of conidial germination kinetics between both fungi would have provided valuable insights; however, the experimental design of the present study did not allow such an analysis. These differences in conidial surface properties may contribute to the reduced phagocytosis rates observed for *S. apiospermum* compared with *A. fumigatus* during the early stages of host-fungus interaction.

The cell walls of *Scedosporium* and *Aspergillus* display several notable differences in polysaccharide composition. Only a few studies have focused on the *Scedosporium* cell-wall determinants involved in recognition by innate immune cells, revealing a role for specific components not detected in *A. fumigatus* [18]. In particular, peptidorhamnomannans are involved in the adhesion of various *Scedosporium* species to epithelial cells, and in the phagocytosis and TNF-α production by macrophages induced by TLR4 activation [32,50]. *Scedosporium* glucosylceramides have also attracted considerable attention, due to their role as important PAMPs interacting with macrophages [51,52]. The pathways highlighted here will pave the way for future studies to identify the *Scedosporium* cell-wall components responsible for the intense inflammatory response observed.

Finally, the highly inflammatory macrophage response triggered by *S. apiospermum* may be linked to different outcomes following human exposure. Strong host immune responses are associated with enhanced fungicidal mechanisms against *S. apiospermum* and lower resistance to macrophage killing, contributing to a lower virulence of *S. apiospermum* and a lower prevalence of *Scedosporium* infections than of *Aspergillus* infections. Conversely, exacerbated inflammatory responses may be deleterious to the host in the long term, particularly in the setting of cystic fibrosis where chronic airway inflammation has been strongly associated with disease severity, progressive lung damage, airway remodeling, and declining pulmonary function [53]. Increased levels of inflammatory markers, including neutrophils, IL-8 and neutrophil elastase in sputum, have been negatively correlated with FEV1 in both pediatric [54] and adult [55] CF populations. Finally, in the context of ABPM, where persistent fungal colonization triggers an exaggerated type‑2 immune response, the beneficial effects of immunomodulatory therapies and the reduction in airway inflammation observed in patients receiving CFTR modulators further support the hypothesis that limiting excessive fungus-induced inflammation may contribute to improved respiratory outcomes [56].

In conclusion, both transcriptomic and phenotypic approaches demonstrated that *S. apiospermum* induces a stronger and earlier inflammatory response in macrophages than *A. fumigatus*. This enhanced inflammatory response may facilitate fungal clearance but could also contribute to the persistence of airway inflammation if CF patients are chronically exposed to *Scedosporium*.

## Supplementary Material

Supplementary materials_Guegan et al.docx

## Data Availability

The data that support the findings of this study are openly available in Zenodo at https://doi.org/10.5281/zenodo.20820703 [57].

## References

[1] Ramirez-Garcia A, Pellon A, Rementeria A, et al. *Scedosporium* and *Lomentospora*: an updated overview of underrated opportunists. Med Mycol. 2018;56(suppl_1):S102–15. doi: 10.1093/mmy/myx113 29538735

[2] Sedlacek L, Graf B, Schwarz C, et al. Prevalence of *Scedosporium* species and *Lomentospora prolificans* in patients with cystic fibrosis in a multicenter trial by use of a selective medium. J Cyst Fibros. 2015;14(2):237–241. doi: 10.1016/j.jcf.2014.12.014 25595044

[3] Cimon B, Carrère J, Vinatier JF, et al. Clinical significance of *Scedosporium apiospermum* in patients with cystic fibrosis. Eur J Clin Microbiol Infect Dis. 2000;19(1):53–56. doi: 10.1007/s100960050011 10706182

[4] Blyth CC, Middleton PG, Harun A, et al. Clinical associations and prevalence of *Scedosporium* spp. in Australian cystic fibrosis patients: identification of novel risk factors? Med Mycol. 2010;48(O1):S37–44. doi: 10.3109/13693786.2010.500627 21067328

[5] Cortez KJ, Roilides E, Quiroz-Telles F, et al. Infections caused by *Scedosporium* spp. Clin Microbiol Rev. 2008;21(1):157–197. doi: 10.1128/CMR.00039-07 18202441 PMC2223844

[6] Bronnimann D, Garcia-Hermoso D, Dromer F, et al. French mycoses study group, characterization of the isolates at the NRCMA. Scedosporiosis/lomentosporiosis observational study (SOS): clinical significance of Scedosporium species identification. Med Mycol. 2021;59(5):486–497. doi: 10.1093/mmy/myaa086 33037432

[7] Cornillet A, Camus C, Nimubona S, et al. Comparison of epidemiological, clinical, and biological features of invasive aspergillosis in neutropenic and non neutropenic patients: a 6-year survey. Clin Infect Dis. 2006;43(5):577–584. doi: 10.1086/505870 16886149

[8] Bertuzzi M, Hayes GE, Icheoku UJ, et al. Anti-*Aspergillus* activities of the respiratory epithelium in health and disease. J Fungi. 2018;4(1):8. doi: 10.3390/jof4010008 PMC5872311 29371501

[9] Latgé J-P, Chamilos G. *Aspergillus fumigatus* and aspergillosis in 2019. Clin Microbiol Rev. 2019;33(1):e00140–18. doi: 10.1128/CMR.00140-18 31722890 PMC6860006

[10] Romani L. Immunity to fungal infections. Nat Rev Immunol. 2011;11(4):275–288. doi: 10.1038/nri2939 21394104

[11] Ibrahim-Granet O, Philippe B, Boleti H, et al. Phagocytosis and intracellular fate of *Aspergillus fumigatus* conidia in alveolar macrophages. Infect Immun. 2003;71(2):891–903. doi: 10.1128/IAI.71.2.891-903.2003 12540571 PMC145364

[12] Goyal S, Castrillón-Betancur JC, Klaile E, et al. The interaction of human pathogenic Fungi with C-Type lectin receptors. Front Immunol. 2018;9:1261. doi: 10.3389/fimmu.2018.01261 29915598 PMC5994417

[13] Beauvais A, Latgé J-P. Special issue: fungal cell wall. J Fungi. 2018;4(3):91. doi: 10.3390/jof4030091 PMC6162784 30081570

[14] Rollin-Pinheiro R, da S XM, Rochetti VP, et al. *Scedosporium* cell wall: from carbohydrate-containing structures to host–pathogen interactions. Mycopathologia. 2020;185(6):931–946. doi: 10.1007/s11046-020-00480-7 32990888

[15] Nosanchuk JD, Casadevall A. The contribution of melanin to microbial pathogenesis. Cell Microbiol. 2003;5(4):203–223. doi: 10.1046/j.1462-5814.2003.00268.x 12675679

[16] van de Veerdonk FL, Gresnigt MS, Romani L, et al. *Aspergillus fumigatus* morphology and dynamic host interactions. Nat Rev Microbiol. 2017;15(11):661–674. doi: 10.1038/nrmicro.2017.90 28919635

[17] de MTP, Aor AC, de OS, et al. Conidial germination in *Scedosporium apiospermum, S. aurantiacum, S. minutisporum* and *Lomentospora prolificans*: influence of growth conditions and antifungal susceptibility profiles. Mem Inst Oswaldo Cruz. 2016;(7):0. doi: 10.1590/0074-02760160200 PMC4957502 27355215

[18] Latgé J-P, Beauvais A, Chamilos G. The cell wall of the human fungal pathogen *Aspergillus fumigatus*: biosynthesis, organization, immune response, and virulence. Annu Rev Microbiol. 2017;71(1):99–116. doi: 10.1146/annurev-micro-030117-020406 28701066

[19] Ghamrawi S, Gastebois A, Zykwinska A, et al. A multifaceted study of *Scedosporium boydii* cell wall changes during germination and identification of GPI-Anchored proteins. PLoS One. 2015;10(6):e0128680. doi: 10.1371/journal.pone.0128680 26038837 PMC4454578

[20] Gersuk GM, Underhill DM, Zhu L, et al. Dectin-1 and TLRs permit macrophages to distinguish between different *Aspergillus fumigatus* cellular states. J Immunol. 2006;176(6):3717–3724. doi: 10.4049/jimmunol.176.6.3717 16517740

[21] Ghamrawi S, Rénier G, Saulnier P, et al. Cell wall modifications during conidial maturation of the human pathogenic fungus *Pseudallescheria boydii*. PLoS One. 2014;9(6):e100290. doi: 10.1371/journal.pone.0100290 24950099 PMC4065047

[22] Buldain I, Martin-Souto L, Antoran A, et al. The host immune response to *Scedosporium/Lomentospora*. J Fungi. 2021;7(2):75. doi: 10.3390/jof7020075 PMC7912657 33499053

[23] Nierman WC, Pain A, Anderson MJ, et al. Genomic sequence of the pathogenic and allergenic filamentous fungus *Aspergillus fumigatus*. Nature. 2005;438(7071):1151–1156. doi: 10.1038/nature04332 16372009

[24] Vandeputte P, Ghamrawi S, Rechenmann M, et al. Draft genome sequence of the pathogenic fungus *Scedosporium apiospermum*. Genome Announc. 2014;2(5):e00988–14. doi: 10.1128/genomeA.00988-14 25278533 PMC4183877

[25] Guegan H, Poirier W, Ravenel K, et al. Deciphering the role of PIG1 and DHN-Melanin in *Scedosporium apiospermum* conidia. J Fungi. 2023;9(2):134. doi: 10.3390/jof9020134 PMC9965090 36836250

[26] Guegan H. Facteurs de virulence de champignons filamenteux au cours de la mucoviscidose: l’exemple de la mélanine [Internet]. 2023; Available from: https://theses.hal.science/tel-04436060v1

[27] Guegan H, Ory K, Belaz S, et al. In vitro and in vivo immunomodulatory properties of octyl-β-d-galactofuranoside during *Leishmania donovani* infection. Parasit Vectors. 2019;12(1):600. doi: 10.1186/s13071-019-3858-0 31870416 PMC6929453

[28] Gibson JF, Johnston SA. Immunity to *Cryptococcus neoformans* and *C.* *gattii* during cryptococcosis. Fungal Genet Biol. 2015;78:76–86. doi: 10.1016/j.fgb.2014.11.006 25498576 PMC4503824

[29] Richardson JP, Moyes DL. Adaptive immune responses to *Candida albicans* infection. Virulence. 2015;6(4):327–337. doi: 10.1080/21505594.2015.1004977 25607781 PMC4601188

[30] Bojang E, Ghuman H, Kumwenda P, et al. Immune sensing of *Candida albicans*. J Fungi. 2021;7(2):119. doi: 10.3390/jof7020119 PMC7914548 33562068

[31] Briard B, Karki R, Malireddi RKS, et al. Fungal ligands released by innate immune effectors promote inflammasome activation during *Aspergillus fumigatus* infection. Nat Microbiol. 2019;4(2):316–327. doi: 10.1038/s41564-018-0298-0 30510167 PMC6619501

[32] Figueiredo RT, Fernandez PL, Dutra FF, et al. TLR4 recognizes *Pseudallescheria boydii* conidia and purified rhamnomannans. J Biol Chem. 2010;285(52):40714–40723. doi: 10.1074/jbc.M110.181255 20959459 PMC3003371

[33] Figari I, Mori N, Palladino MJ. Regulation of neutrophil migration and superoxide production by recombinant tumor necrosis factors-alpha and -beta: comparison to recombinant interferon-gamma and interleukin-1 alpha. Blood. 1987;70(4):979–984. doi: 10.1182/blood.V70.4.979.979 2820532

[34] Saïd-Sadier N, Padilla E, Langsley G, et al. *Aspergillus fumigatus* stimulates the NLRP3 inflammasome through a pathway requiring ROS production and the Syk tyrosine kinase. PLoS One. 2010;5(4):e10008. doi: 10.1371/journal.pone.0010008 20368800 PMC2848854

[35] Ketelut-Carneiro N, Silva GK, Rocha FA, et al. IL-18 triggered by the Nlrp3 inflammasome induces host innate resistance in a pulmonary model of fungal infection. J Immunol. 2015;194(9):4507–4517. doi: 10.4049/jimmunol.1402321 25825440

[36] van de Veerdonk FL, Netea MG, Dinarello CA, et al. Inflammasome activation and IL-1β and IL-18 processing during infection. Trends Immunol. 2011;32(3):110–116. doi: 10.1016/j.it.2011.01.003 21333600

[37] Zelante T, De Luca A, D’Angelo C, et al. IL-17/Th17 in anti-fungal immunity: What’s new? Eur J Immunol. 2009;39(3):645–648. doi: 10.1002/eji.200839102 19283705

[38] Mezger M, Steffens M, Beyer M, et al. Polymorphisms in the chemokine (C-X-C motif) ligand 10 are associated with invasive aspergillosis after allogeneic stem-cell transplantation and influence CXCL10 expression in monocyte-derived dendritic cells. Blood. 2008;111(2):534–536. doi: 10.1182/blood-2007-05-090928 17957030

[39] Mello TP, Oliveira SSC, Frasés S, et al. Surface properties, adhesion and biofilm formation on different surfaces by *Scedosporium* spp. and *Lomentospora prolificans*. Biofouling. 2018;34(7):800–814. doi: 10.1080/08927014.2018.1503652 30354689

[40] Caneppa A, de Meirelles JV, Rollin-Pinheiro R, et al. Structural differences influence biological properties of glucosylceramides from Clinical and environmental isolates of *Scedosporium aurantiacum* and *pseudallescheria minutispora*. J Fungi. 2019;5(3):62. doi: 10.3390/jof5030062 PMC6787682 31311197

[41] Mello TP, Branquinha MH, Santos ALS. Biofilms formed by *Scedosporium* and *Lomentospora* species: focus on the extracellular matrix. Biofouling. 2020;36(3):308–318. doi: 10.1080/08927014.2020.1759558 32401558

[42] Rollin-Pinheiro R, de Meirelles JV, Vila TVM, et al. Biofilm formation by *Pseudallescheria/Scedosporium* species: a comparative study. Front Microbiol. 2017;8:1568. doi: 10.3389/fmicb.2017.01568 28868050 PMC5563321

[43] Mahmoud DE, Hanachi M, Yaakoub H, et al. Functional insights into human macrophage response against *Scedosporium apiospermum* and *Scedosporium dehoogii*. Cytokine. 2023;172:156384. doi: 10.1016/j.cyto.2023.156384 37832161

[44] Warris A, Netea MG, Verweij PE, et al. Cytokine responses and regulation of interferon-gamma release by human mononuclear cells to *Aspergillus fumigatus* and other filamentous fungi. Med Mycol. 2005;43(7):613–621. doi: 10.1080/13693780500088333 16396246

[45] Tronchin G, Pihet M, Lopes-Bezerra LM, et al. Adherence mechanisms in human pathogenic fungi. Med Mycol. 2008;46(8):749–772. doi: 10.1080/13693780802206435 18651303

[46] Dague E, Alsteens D, Latgé J-P, et al. High-resolution cell surface dynamics of germinating *Aspergillus fumigatus* conidia. Biophys J. 2008;94(2):656–660. doi: 10.1529/biophysj.107.116491 17890393 PMC2157220

[47] Thau N, Monod M, Crestani B, et al. Rodletless mutants of *Aspergillus fumigatus*. Infect Immun. 1994;62(10):4380–4388. doi: 10.1128/iai.62.10.4380-4388.1994 7927699 PMC303120

[48] Bouchara JP, Sanchez M, Esnault K, et al. Interactions between *Aspergillus fumigatus* and host matrix proteins. Contrib Microbiol. 1999;2:167–181. 10523273 10.1159/000060293

[49] Tronchin G, Esnault K, Renier G, et al. Expression and identification of a laminin-binding protein in *Aspergillus fumigatus* conidia. Infect Immun. 1997;65(1):9–15. doi: 10.1128/iai.65.1.9-15.1997 8975886 PMC174550

[50] de Meirelles JV, Xisto MIDDS, da S XM, et al. Peptidorhamanomannan: A surface fungal glycoconjugate from *Scedosporium aurantiacum* and *Scedosporium minutisporum* and its recognition by macrophages. Med Mycol. 2021;59(5):441–452. doi: 10.1093/mmy/myaa065 32766889

[51] Rochetti VP, Rollin-Pinheiro R, de Oliveira EB, et al. Glucosylceramide plays a role in fungal germination, lipid raft organization and biofilm adhesion of the pathogenic fungus *Scedosporium aurantiacum*. J Fungi. 2020;6(4):E345. doi: 10.3390/jof6040345 PMC7762401 33302332

[52] Rollin-Pinheiro R, Liporagi-Lopes LC, de Meirelles JV, et al. Characterization of *Scedosporium apiospermum* glucosylceramides and their involvement in fungal development and macrophage functions. PLoS One. 2014;9(5):e98149. doi: 10.1371/journal.pone.0098149 24878570 PMC4039464

[53] Poore TS, Hong G, Zemanick ET. Fungal infection and inflammation in cystic fibrosis. Pathogens. 2021;10(5):618. doi: 10.3390/pathogens10050618 34069863 PMC8157353

[54] Sagel SD, Sontag MK, Wagener JS, et al. Induced sputum inflammatory measures correlate with lung function in children with cystic fibrosis. J Pediatr. 2002;141(6):811–817. doi: 10.1067/mpd.2002.129847 12461498

[55] Mayer-Hamblett N, Aitken ML, Accurso FJ, et al. Association between pulmonary function and sputum biomarkers in cystic fibrosis. Am J Respir Crit Care Med. 2007;175(8):822–828. doi: 10.1164/rccm.200609-1354OC 17234902 PMC2720115

[56] Chatterjee P, Moss CT, Omar S, et al. Allergic bronchopulmonary aspergillosis (ABPA) in the Era of cystic fibrosis transmembrane conductance regulator (CFTR) modulators. J Fungi. 2024;10(9):656. doi: 10.3390/jof10090656 PMC11433030 39330416

[57] Guegan H. Early inflammatory macrophage reprogramming by *Scedosporium apiospermum* determines fungal survival [Internet]. 2026; Available from: https://zenodo.org/doi/10.5281/zenodo.20820703 10.1080/21505594.2026.2707829 PMC13540164 42683833

