# Supplementary material for: Early inflammatory macrophage reprogramming by Scedosporium apiospermum determines fungal survival
Source: Virulence. 2026 Sep 2;17(1):2707829. doi: 10.1080/21505594.2026.2707829 (PMC13540164; doi:10.1080/21505594.2026.2707829)
Supplement: Supplementary file 1 [file KVIR_A_2707829_SM8310.docx]

**Supplementary materials**

**Table S1. Nucleotide sequence primers used for RT-qPCR on human macrophages**

| Gene target | Forward primer (5’-3’) | Reverse primer (5’-3’) |
| --- | --- | --- |
| ACTB | CGAGATCATGTTTGAGACCT | GGCATACCCCTCGTAGAT |
| GAPDH | ATGACATCAAGAAGGTGGTG | CATACCAGGAAATGAGCTTG |
| IFN-γ | TGTCCAACGCAAAGCAATAC | ATATTGCAGGCAGGACAACC |
| TN-Fα | AACCTCCTCTCTGCCATCAA | ATGTTCGTCCTCCTCACAGG |
| IL-6 | AGTACCCCCAGGAGAAGAT | ATCTTTGGAAGGTTCAGGTT |
| IL-1β | TCATGCTCTGTTCTTGGGAAT | GCTTGTCCTGCTTTCTGTTC |
| STAT4 | GGATTGAAAATCAAGACTGG | GAAATAACCACAGCTACATGC |
| CXCL9 | TGTCTAAACCCAGATTCAGC | GTCTTCTTTTGACGAGAACG |
| CCL15 | CCAGTTCATAAATGATGCAG | GCTGCTCGTTTCAAAATAAC |
| CCR2 | GGCCTGAGTAACTGTGAAAG | CGAGGAGTCCTTGTGTAGTC |
| SOC3 | GCTCCAAGAGCGAGTACC | CTGAGCGTGAAGAAGTGG |

**Table S2. List of genes differentially expressed with |log_2_FC |>2 in Sap-infected macrophages relative to Afu-infected macrophages, 4 h and 12 h postinfection**

| **Gene symbol** | **Gene ID** | **Gene name** | | | **Log_2_FC** | | | **FDR** | | | |  |  |
| --- | --- | --- | --- | --- | --- | --- | --- | --- | --- | --- | --- | --- | --- |
| **4 h** | | | | | | | | | | | | | |
| NA | NA | | NA | | | 3.615979772 | | | 9.7713E-03 | | | |  |
| IL12B | 3593 | | interleukin 12B | | | 3.474519546 | | | 1.0081E-03 | | | |  |
| CCL15 | 6359 | | C-C motif chemokine ligand 15 | | | 3.397041087 | | | 6.7596E-04 | | | |  |
| CSF2 | 1437 | | Colony-stimulating factor 2 | | | 2.995533821 | | | 1.2607E-13 | | | |  |
| HHLA2 | 11148 | | HERV-H LTR-associating 2 | | | 2.982991889 | | | 1.5564E-03 | | | |  |
| C2CD4B | 388125 | | C2 calcium-dependent domain-containing 4B | | | 2.822515759 | | | 1.0374E-03 | | | |  |
| NA | NA | | NA | | | 2.696077301 | | | 7.1240E-04 | | | |  |
| SHF | 90525 | | Src homology 2 domain-containing F | | | 2.621231596 | | | 1.5683E-07 | | | |  |
| IL6 | 3569 | | interleukin 6 | | | 2.597115641 | | | 4.0563E-08 | | | |  |
| IL36G | 56300 | | interleukin 36 gamma | | | 2.539323017 | | | 3.1051E-07 | | | |  |
| C2CD4A | 145741 | | C2 calcium-dependent domain-containing 4A | | | 2.536819841 | | | 3.6509E-02 | | | |  |
| PTGS2 | 5743 | | Prostaglandin endoperoxide synthase 2 | | | 2.50294111 | | | 1.0730E-15 | | | |  |
| CSF3 | 1440 | | Colony-stimulating factor 3 | | | 2.486916793 | | | 5.4509E-07 | | | |  |
| IL1B | 3553 | | interleukin 1 beta | | | 2.319588337 | | | 7.3563E-08 | | | |  |
| RN7SL124P | 106480955 | | RNA, 7SL, cytoplasmic 124, pseudogene | | | 2.255663769 | | | 1.8383E-02 | | | |  |
| IL10 | 3586 | | interleukin 10 | | | 2.220330931 | | | 1.1588E-03 | | | |  |
| SERPINB2 | 5055 | | serpin family B member 2 | | | 2.217195465 | | | 2.1187E-06 | | | |  |
| EREG | 2069 | | epiregulin | | | 2.161912859 | | | 1.8966E-13 | | | |  |
| MACC1-DT | 100506098 | | MACC1 divergent transcript | | | 2.123239493 | | | 1.3793E-02 | | | |  |
| NR4A2 | 4929 | | nuclear receptor subfamily 4 group A member 2 | | | 2.101521576 | | | 5.0988E-08 | | | |  |
| UPB1 | 51733 | | beta-ureidopropionase 1 | | | 2.045897752 | | | 2.0125E-03 | | | |  |
| ADORA2A | 135 | | adenosine A2a receptor | | | 2.005383369 | | | 1.5570E-04 | | | |  |
| IER3 | 8870 | | immediate early response 3 | | | 2.001262757 | | | 1.0511E-08 | | | |  |
| CXCL9 | 4283 | | C-X-C motif chemokine ligand 9 | | | -3.130474419 | | | 1.1010E-05 | | | |  |
| CXCL10 | 3627 | | C-X-C motif chemokine ligand 10 | | | -2.573159468 | | | 3.3086E-03 | | | |  |
| BATF2 | 116071 | | basic leucine zipper ATF-like transcription factor 2 | | | -2.139447643 | | | 4.9695E-04 | | | |  |
| CCL8 | 6355 | | C-C motif chemokine ligand 8 | | | -2.062748476 | | | 6.7030E-03 | | | |  |
| **12 h** | | | | | | | | | | | | | |
| IGFBP4 | 3487 | | | Insulin-like growth factor-binding protein 4 | | 3.659827772 | | | | 3.6783E-07 | | | |
| NA | NA | | | NA | | 3.597599163 | | | | 3.4890E-07 | | | |
| IL36G | 56300 | | | interleukin 36 gamma | | 3.14324156 | | | | 2.2402E-10 | | | |
| HSPA1B | 3304 | | | Heat-shock protein family A (Hsp70) member 1B | | 2.894631431 | | | | 2.5359E-13 | | | |
| SEMA3F | 6405 | | | semaphorin 3F | | 2.850735327 | | | | 4.2856E-08 | | | |
| TM4SF1 | 4071 | | | transmembrane 4 L six family member 1 | | 2.682821042 | | | | 1.3260E-04 | | | |
| IL12B | 3593 | | | interleukin 12B | | 2.661134722 | | | | 1.2666E-03 | | | |
| PDCD1 | 5133 | | | programmed cell death 1 | | 2.58773484 | | | | 3.4976E-02 | | | |
| ATP1B2 | 482 | | | ATPase Na^+^/K^+^-transporting subunit beta 2 | | 2.48605068 | | | | 2.2755E-06 | | | |
| THBD | 7056 | | | thrombomodulin | | 2.390763262 | | | | 2.3561E-11 | | | |
| CD300E | 342510 | | | CD300e molecule | | 2.360336557 | | | | 7.6056E-08 | | | |
| MMP1 | 4312 | | | matrix metallopeptidase 1 | | 2.32499619 | | | | 5.4831E-10 | | | |
| DUSP4 | 1846 | | | Dual-specificity phosphatase 4 | | 2.28399713 | | | | 2.3371E-15 | | | |
| CCL15 | 6359 | | | C-C motif chemokine ligand 15 | | 2.201899119 | | | | 4.0701E-03 | | | |
| LINC00659 | 100652730 | | | long intergenic non-protein coding RNA 659 | | 2.157625659 | | | | 2.5506E-06 | | | |
| ADA | 100 | | | adenosine deaminase | | 2.120694609 | | | | 3.8291E-08 | | | |
| LOXL2 | 4017 | | | lysyl oxidase like 2 | | 2.111804062 | | | | 9.7290E-07 | | | |
| DUSP15 | 128853 | | | Dual-specificity phosphatase 15 | | 2.060833669 | | | | 7.1566E-04 | | | |
| C2CD4B | 388125 | | | C2 calcium-dependent domain-containing 4B | | 2.044172222 | | | | 3.8374E-05 | | | |
| CCL1 | 6346 | | | C-C motif chemokine ligand 1 | | 2.010707968 | | | | 1.4541E-02 | | | |
| Continuation of Table S2 | | | | | | | | | | | | | |
| **Gene symbol** | **Gene ID** | | | **Gene name** | | **Log_2_FC** | | | | **FDR** | | | |
| CXCL9 | 4283 | | | C-X-C motif chemokine ligand 9 | | -3.745486246 | | | | 5.4935E-10 | | | |
| FRMD7 | 90167 | | | FERM domain-containing 7 | | -3.272028896 | | | | 1.5127E-06 | | | |
| NA | NA | | | NA | | -3.12075105 | | | | 4.7662E-05 | | | |
| CXCL10 | 3627 | | | C-X-C motif chemokine ligand 10 | | -3.097327477 | 1.2410E-04 | | | |  |  |  |
| EGR4 | 1961 | | | early growth response 4 | | -3.083346969 | 1.3330E-08 | | | |  |  |  |
| LINC02015 | 102724550 | | | long intergenic non-protein coding RNA 2015 | | -2.803877347 | 2.3861E-05 | | | |  |  |  |
| ARHGAP31-AS1 | 100874246 | | | ARHGAP31 antisense RNA 1 | | -2.742747557 | 3.2490E-06 | | | |  |  |  |
| NA | NA | | | NA | | -2.72531489 | 4.1850E-02 | | | |  |  |  |
| H1-4 | 3008 | | | H1.4 linker histone, cluster member | | -2.627323954 | 3.6442E-06 | | | |  |  |  |
| NA | NA | | | NA | | -2.456860766 | 6.6611E-05 | | | |  |  |  |
| HAPLN3 | 145864 | | | hyaluronan and proteoglycan link protein 3 | | -2.452625313 | 6.7369E-11 | | | |  |  |  |
| C12orf50 | 160419 | | | chromosome 12 open reading frame 50 | | -2.444439477 | 9.0801E-06 | | | |  |  |  |
| GBP1P1 | 400759 | | | guanylate binding protein 1 pseudogene 1 | | -2.434542798 | 1.8971E-05 | | | |  |  |  |
| SECTM1 | 6398 | | | secreted and transmembrane 1 | | -2.331069684 | 5.7361E-10 | | | |  |  |  |
| GRIN3A | 116443 | | | glutamate ionotropic receptor NMDA type subunit 3A | | -2.305839672 | 8.1639E-04 | | | |  |  |  |
| NA | NA | | | NA | | -2.27991136 | 2.9183E-05 | | | |  |  |  |
| ETV7 | 51513 | | | ETS variant transcription factor 7 | | -2.277199573 | 7.7379E-09 | | | |  |  |  |
| CAMK1G | 57172 | | | calcium/calmodulin-dependent protein kinase IG | | -2.261408635 | 1.8550E-03 | | | |  |  |  |
| CSPG4BP | 727930 | | | chondroitin sulfate proteoglycan family member 4B, pseudogene | | -2.24328696 | 3.2014E-07 | | | |  |  |  |
| NA | NA | | | NA | | -2.239271345 | 1.4739E-05 | | | |  |  |  |
| LINC02528 | 105378020 | | | long intergenic non-protein coding RNA 2528 | | -2.22357937 | 7.4276E-04 | | | |  |  |  |
| KIF25-AS1 | 100505879 | | | KIF25 antisense RNA 1 | | -2.218271315 | 7.1181E-04 | | | |  |  |  |
| GBP5 | 115362 | | | Guanylate-binding protein 5 | | -2.165170262 | 3.8867E-09 | | | |  |  |  |
| PRECSIT | 283487 | | | p53-regulated carcinoma-associated Stat3-activating long intergenic non-protein coding transcript | | -2.136557379 | 5.4308E-08 | | | |  |  |  |
| CIITA | 4261 | | | class II major histocompatibility complex transactivator | | -2.098592007 | 4.6876E-14 | | | |  |  |  |
| FCGR1CP | 100132417 | | | Fc gamma receptor Ic, pseudogene | | -2.088519681 | 3.0509E-09 | | | |  |  |  |
| NA | NA | | | NA | | -2.075685623 | 6.3551E-05 | | | |  |  |  |
| LINC01215 | 101929623 | | | long intergenic non-protein coding RNA 1215 | | -2.070214262 | 3.4398E-10 | | | |  |  |  |
| CCL8 | 6355 | | | C-C motif chemokine ligand 8 | | -2.036568423 | 4.0944E-03 | | | |  |  |  |
| ISL2 | 64843 | | | ISL LIM homeobox 2 | | -2.024151969 | 4.7719E-04 | | | |  |  |  |
| LOC124908063 | 124908063 | | | uncharacterized LOC124908063 | | -2.013316866 | 4.9205E-03 | | | |  |  |  |

**Supplementary figures**

**Figure S1. Genes differentially expressed in *S. apiospermum* (Sap)-infected macrophages relative to uninfected macrophages.** (a) Volcano plots of DEGs at 4 h and 12 h pi. Green and red dots represent significant DEGs (FDR<0.05) downregulated (log_2_FC <-1) and upregulated (log_2_FC>1), respectively. Light gray and black dots are genes with │log_2_FC │<1 or FDR>0.05. Gene IDs for some of the most highly dysregulated genes are shown. (b) Histograms showing the number of DEGs at 4 h and 12 h, for thresholds of│log_2_FC│ >1 (left) or >2 (right). (c) Venn diagrams showing the number of genes downregulated (left) or upregulated (right) specifically at 4 h or 12 h, or at both time points in infection (│log_2_FC│ >1).

**Figure S2. Genes differentially expressed between *A. fumigatus* (Afu)-infected macrophages and uninfected macrophages.** (a) Volcano plots of DEGs at 4 h and 12 h pi. Green and red dots represent significant DEGs (FDR<0.05), downregulated (log_2_FC <-1) and upregulated (log_2_FC>1), respectively. Light gray and black dots correspond to genes with │log_2_FC │<1 or FDR>0.05. Gene IDs for some of the most highly dysregulated genes are shown. (b) Histograms showing the number of DEGs at 4 h and 12 h, for thresholds of│log_2_FC│ >1 (left) or >2 (right). (c) Venn diagrams showing the number of genes which downregulated (left) or upregulated (right) specifically at 4 h or 12 h, or both time points in infection (│log_2_FC│ >1).

**Figure S3. Fold-change in expression of selected genes according to RT-qPCR and RNA-seq.** The data shown are the expression ratio in Sap-infected macrophages relative to Afu-infected macrophages at 4 h postinfection for a selection of DEGs, according to RNA-seq.
